# Monoclonal antibodies and chimeric antigen receptor (CAR) T cells in the treatment of colorectal cancer

**DOI:** 10.1186/s12935-021-01763-9

**Published:** 2021-02-01

**Authors:** Ke-Tao Jin, Bo Chen, Yu-Yao Liu, H uan-Rong Lan, Jie-Ping Yan

**Affiliations:** 1 Department of Colorectal Surgery, Affiliated Jinhua Hosptial, Zhejiang University School of Medicine, Zhejiang Province, Jinhua, 312000 P.R. China; 2 Department of Neurology, Zhejiang Provincial People’s Hospital, People’s Hospital of Hangzhou Medical College, Hangzhou, 310014 China; 3 Department of Breast and Thyroid Surgery, Affiliated Jinhua Hosptial, Zhejiang University School of Medicine, Zhejiang Province, Jinhua, 312000 P.R. China; 4 Department of Pharmacy, Zhejiang Provincial People’s Hospital, People’s Hospital of Hangzhou Medical College, No. 158 Shangtang Road, Hangzhou, 310014 China

**Keywords:** Colorectal cancer, Immunotherapy, Monoclonal antibody, Chimeric antigen receptor (CAR) T cells

## Abstract

Colorectal cancer (CRC) is the third most common cancer and the second leading cause of cancer deaths worldwide. Besides common therapeutic approaches, such as surgery, chemotherapy, and radiotherapy, novel therapeutic approaches, including immunotherapy, have been an advent in CRC treatment. The immunotherapy approaches try to elicit patients` immune responses against tumor cells to eradicate the tumor. Monoclonal antibodies (mAbs) and chimeric antigen receptor (CAR) T cells are two branches of cancer immunotherapy. MAbs demonstrate the great ability to completely recognize cancer cell-surface receptors and blockade proliferative or inhibitory pathways. On the other hand, T cell activation by genetically engineered CAR receptor via the TCR/CD3 and costimulatory domains can induce potent immune responses against specific tumor-associated antigens (TAAs). Both of these approaches have beneficial anti-tumor effects on CRC. Herein, we review the different mAbs against various pathways and their applications in clinical trials, the different types of CAR-T cells, various specific CAR-T cells against TAAs, and their clinical use in CRC treatment.

## Background

Colorectal cancer (CRC) is the second leading cause of cancer death (about 881,000 deaths) and the third most common malignancy (about 1.8 million new cases) in the world [1, 2]. CRC is a multifactorial disease resulting from genetic alterations [3], diet, lifestyle [4], exposure to environmental xenobiotics [5], and intestinal microbiota [6]. Despite enormous improvements in the strategies of CRC treatment, including chemotherapy, radiotherapy, surgery, and nutritional-supplement therapy [7], the prognosis of patients with metastatic CRC remains poor, with a median overall survival (OS) of about 30 months [8]. For this reason, the development of novel and more effective therapeutic approaches are necessary for metastatic CRC. Owing to the suitable tumor microenvironment (TME) in CRC for expressing molecular markers and receptors such as VEGF (vascular endothelial growth factor) [9], EGFR (epidermal growth factor receptor) [10], IGF-1R (insulin-like growth factor 1 receptor) [11], HER2/neu (human epidermal growth factor receptor-2) [12], α_V_β_3_ integrin [13], MUC5AC (mucin 5AC) [14], DR5 (death receptor) [15], CTLA-4 (cytotoxic T-lymphocyte associated protein 4), and PD-L1 (programmed death-ligand 1) [16], compared with healthy cells, immunotherapy strategies using monoclonal antibodies (mAbs) and chimeric antigen receptor (CAR) T cells are promising therapeutic candidates in CRC treatment. MAbs display important ability to completely identify and classify cancer cell-surface receptors, while CAR T-cells are activated upon antigen engagement to counteract physiologic response suppression and enhancement the therapeutic efficacy against cancers. In this paper, we review the development of two novel therapeutic approaches, mAbs and CAR T-cells, in CRC treatment. We also summarize the available data with the use mAbs and CAR T-cells as well as their challenges and hopes.

## Immune cells and responses in the CRC

The immune system has a multi-faceted and complex role in CRC, affecting its all aspect from tumorigenesis to treatment. Immune cells can act as tumor promotors by inducing tumor cell proliferation and metastasis, as well as tumor suppressors by inhibiting tumor initiation and progression. Various immune cells within the TME, including macrophages, dendritic cells (DCs), neutrophils, mast cells, natural killer (NK) cells, myeloid-derived suppressor cells (MDSCs), and B and T lymphocytes, interact with tumor cells (directly or through cytokine and chemokine signals), shaping the tumor cells behavior and response to treatment [17]. An algorithmic study revealed 22 immune cell types in the TME of CRC patients, which were heterogenic in different tumor stages [18].

It has been shown that infiltration of M1 macrophages, T follicular helper (T-fh) cells, Th1 cells, NK cells, and DCs are associated with a good prognosis in CRC patients, whereas a poor outcome is associated with MDSCs, B cells, Th17, and M2 macrophages [19]. Immunosuppressive cells inhibit immune responses against tumor cells, leading to tumor progression and development. For example, MDSCs can suppress anti-tumor activities of NK cells and T cells. Furthermore, they establish pre-metastasis niches, promote angiogenesis, and recruit other immunosuppressive cells, such as regulatory T cells [20]. Hu et al*.* found that CD39^+^ γδT cells, as immunosuppressive T cells, significantly increased in the CRC tissues and highly expressed immunosuppression-related molecules, including CD25, CD161, FOXP3, programmed cell death protein 1 PD-1, CTLA-4, PD-L1, whereas they expressed markedly lower levels of immunostimulatory factors [21]. Also, M2 macrophages can mediate resistance to chemotherapy, tumor cell migration and invasion, and angiogenesis [22–24]. Moreover, it has been shown that the gut microbiome can affect the immune responses in CRC patients. For instance, *Fusobacterium nucleatum* inhibits T cell proliferation and increases T cell apoptosis by expanding MDSCs [6, 25]. Thus, understanding immune cells in TME and their interaction with tumor cells allows scientists to identify, develop, and individualize novel immune-based therapeutic agents in CRC patients.

## Monoclonal antibodies

### Overview of monoclonal antibodies

**Fig. 1:**
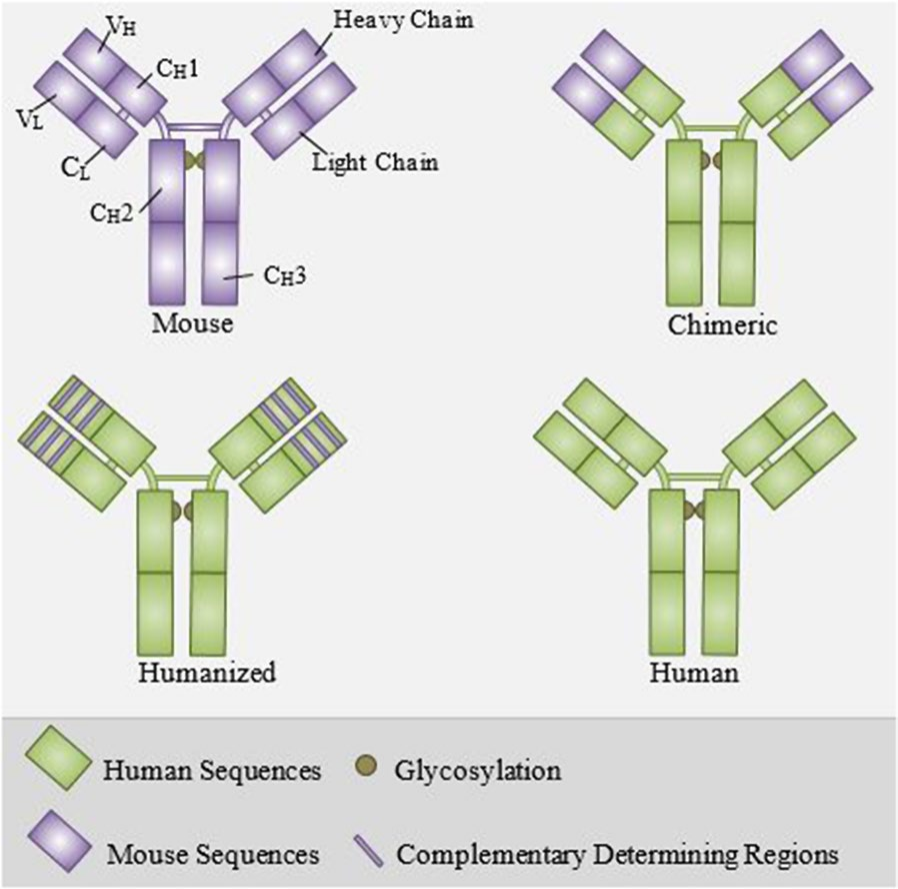
The structure of different types of mAbs. All the components of mouse mAbs are derived from mice. In chimeric mAbs, the variable regions of a mice Ab is fused with the constant regions of a human Ab. In humanized mAbs, only the hypervariable regions (CDRs) of the mAb are originated from mice. All the components of human mAbs are derived from humans

Whilst antibodies (Abs) secreted by B-cells in response to and neutralizing an antigen are usually polyclonal, Kohler and Milstein produced murine mAbs from hybridomas in 1975 [26]. Although murine mAbs were developed for clinical application, allergic reactions, the induction of anti-drug antibodies (ADAs), and short half-life in humans shifted the technology toward chimeric mouse-human and then humanized Abs [27]. In the chimeric mouse-human Abs, the entire variable regions of a mice Ab is fused with the constant regions of a human Ab to reduce immunogenicity and extend the half-life, but the tendency of chimeric mAbs to induce ADAs is still substantial [28]. The next generation of mAbs was humanized ones in which only the hypervariable regions (complementarity determining regions/CDRs) of the mAb are originated from mice [29]. Fully human mAbs are state of the art in the construction of mAbs which are produced in transgenic mice carrying the human immunoglobulin locus [30]. The structure of different types of mAbs is presented in Fig. 1.

Over the past decades, the significance of therapeutic mAbs has been dramatically increased due to their efficacy in the treatment of different diseases. Muromonab-CD3, as the first therapeutic mAb, was approved against T cell-expressed CD3 for the prevention of acute transplant rejection in 1986 [31]. Until the late 1990s, the growth of approved mAbs was slow, but the advent of chimeric, humanized, and fully human mAbs increased the rate of approval and sales of mAb products. Until 2019, the US FDA has approved 79 therapeutic mAbs, including 30 mAbs for cancer treatment [32]. Besides recombinant proteins, mAbs are the foremost leaders of the biopharmaceutical market [33]. Sales of mAbs showed a 90% increase between 2008 and 2013 from $39 billion to $75 billion [31], and according to forecasts, Ab-based medicine will occupy 20% of the pharmaceutical market in 2022, with sales of $172.8 billion [33].

### Monoclonal antibodies for CRC therapy

It has been shown that molecularly targeted agents are more efficient for improving OS and progression-free survival (PFS) of metastatic CRC [34]. For this reason, several molecularly targeted approaches are developed for first- and second-line treatment in combination with chemotherapy regimens (FOLFOX, FOLFIRI, and FOLFOXIRI) [8, 35]. For example, bevacizumab (anti-VEGF), cetuximab, and panitumumab (anti-EGFR) are approved for both first- and second-line treatment [8]. In this section, we reviewed the developed mAbs in the (pre)-clinical studies according to their targets. Moreover, Table 1 summarizes the application of mAbs in the treatment of CRC in clinal trials.

**Table 1:** Monoclonal antibodies under clinical trials for the treatment of colorectal cancer

mAb	Target	Phase	Coadministration	NCT number
Bevacizumab	VEGF-A	I/II	FOLFIRINOX3	NCT03795311
Bevacizumab	VEGF-A	II	Xelox/Xeliri	NCT01531595
Ramucirumab	VEGFR2	II	TAS 102	NCT03520946
Aflibercept	VEGF-A,-B, PlGF	II	LV5FU2	NCT02384759
Aflibercept	VEGF-A,-B, PlGF	II	mLV5FU2/ mFOLFOX7	NCT03530267
VGX-100	VEGF-C	I	Bevacizumab	NCT01514123
Gevokizumab	IL-1β	I	Standard therapies	NCT03798626
Cetuximab	EGFR	I/II	MEN1611	NCT04495621
Cetuximab	EGFR	III	FOLFIRI	NCT03391934
Trastuzumab	HER2	II	Tucatinib	NCT03043313
Panitumumab	EGFR	II	FOLFOX,FOLFIRI or irinotecan	NCT03311750
Panitumumab	EGFR	II	Niraparib	NCT03983993
Nivolumab	PD-1	II	FOLFOXIRI/Bevacizumab	NCT04072198
Nivolumab	PD-1	I/II	Guadecitabine	NCT03576963
Pembrolizumab	PD-1	I/II	Regorafenib	NCT03657641
Pembrolizumab	PD-1	I	Grapiprant	NCT03658772
Atezolizumab	PD-L1	II	Bevacizumab	NCT02982694
Durvalumab	PD-L1	II	Trametinib	NCT03428126
Ipilimumab	CTLA-4	II	FOLFOX/ Nivolumab	NCT04430985
Tremelimumab	CTLA-4	I	Durvalumab	NCT02754856
Genolimzumab	PD-1	I	Fruquintinib	NCT03977090

### Vascular endothelial growth factor pathway

VEGF family, as the prominent factor in angiogenesis, consists of six members, including VEGF-A, B, C, D, E, and placental growth factors (PlGF) [36]. VEGF families activate several intracellular signaling by binding to their receptors, including VEGFR1, VEGFR2, and VEGFR3 [37]. During solid tumor growth, cells become hypoxic and need new blood vessels to provide oxygen and nutrients for surviving [38]. It has been shown that the expression of VEGF is increased in several malignancies, including pancreatic, liver, gastric, and colorectal cancer [39]. There is a positive correlation between the expression of VEGF and stage of CRC; VEGF expression is higher in patients with stage IV than patients with stage II and III. Moreover, the expression of VEGF is significantly higher in dead patients compared with those who survived for 10 years [40].

#### Bevacizumab (Avastin®)

Bevacizumab is a humanized mAb that binds to VEGF-A and prevents its interaction with VEGFR, leading to inhibition of VEGF signaling pathways and angiogenesis [41]. Although bevacizumab was first approved for the treatment of metastatic CRC in the combination of chemotherapy in 2004 [42], it is widely recommended for the treatment of various cancers, including breast, ovarian, and lung cancers [43–45]. Several randomized trials confirmed the efficacy of bevacizumab in combination with chemotherapy agents in multiple lines of treatment of patients with CRC [46–48]. Recently, a meta-analysis study was conducted on seven randomized trials to evaluate the effect of bevacizumab in combination with first-line chemotherapy agents in metastatic CRC. The study revealed that the addition of bevacizumab to only the fluoropyrimidine monotherapy regimen could prolong PFS and OS [49].

#### Ramucirumab (Cyramza®)

Ramucirumab, a fully human mAb against the extracellular domain of VEGFR2, was approved by the US FDA for the treatment of metastatic CRC in combination with 5-FU, leucovorin, and irinotecan (FOLFIRI) in the second-line setting [50]. According to two phase I clinical trials using ramucirumab, the recommended doses of the mAb for phase II were established as 8 mg/kg every 2 weeks or 10 mg/kg every 3 weeks [51, 52]. In a phase II study, intravenous administration of ramucirumab in combination with the mFOLFOX-6 regimen prolonged PFS and OS, but some adverse events were observed, including neutropenia, neuropathy, and hypertension [53]. A randomized, double-blind phase III RAISE trial demonstrated that ramucirumab versus placebo in combination with FOLFIRI in patients with metastatic CRC significantly improved OS [54].

#### Aflibercept (Zaltrap®)

Aflibercept, a fully humanized recombinant fusion protein, consists of extracellular domains of VEGFR1 and VEGFR2 fused to the Fc portion of human immunoglobulin G1 which binds to VEGF-A, -B, and PlGF [55]. It has been shown that aflibercept has strong anti-angiogenesis effects compared with bevacizumab owing to longer half-life, higher affinity to soluble VEGFs, and added affinity to PlGF [56]. Multiple phase I trials have demonstrated the safety of aflibercept in combination with chemotherapy regimens in various cancers [57–59]. A phase II clinical trial using aflibercept in combination with FOLFIRI in Japanese patients with CRC showed beneficial effects as a second-line treatment [60]. In a multicenter phase III study, aflibercept combined with FOLFIRI regimen improved survival and response rates in CRC patients treated with prior oxaliplatin-based therapy [61].

#### Tanibirumab

Tanibirumab is a fully human mAb against VEGFR2 which blocks angiogenesis and thereby inhibits tumor growth. It has been shown that tanibirumab has strong anti-tumor effects as a single agent in glioblastoma, breast, and colorectal tumor models [62]. A phase I trial of tanibirumab in patients with refractory solid tumors revealed modest clinical efficacy and tolerable toxicity profile [63].

#### Vanucizumab

Vanucizumab is a bispecific mAb targeting angiopoietin-2 (Ang-2), an angiogenesis growth factor, and VEGF-A [64]. It has been found that vanucizumab inhibits tumor growth, metastasis, and angiogenesis more effectively than monotherapy using mAbs against Ang-2 or VEGF-A in a murine cancer model [64, 65]. A phase I study using biweekly administration of vanucizumab in patients with solid tumors confirmed its safety and anti-angiogenesis activities [66]. Recently, the results of a phase II clinical trial, McCAVE trial, in patients with previously untreated metastatic CRC revealed that angiogenesis inhibition by vanucizumab does not provide additional benefits over bevacizumab for first‐line treatment of metastatic CRC [67].

### Epidermal growth factor receptor pathway

Epidermal growth factor receptor (EGFR) family, transmembrane proteins involved in the cell growth, differentiation, and survival, includes EGFR (HER1), HER2, HER3, and HER4 [68]. Binding of the EGFRs to their ligands, such as EGF, induces homo-/hetero-dimerization of EGFR that triggers the phosphorylation of tyrosine kinases in the intracellular domain of the receptor. Subsequently, the complex signaling network activated via EGFR, such as the PI3K-Akt pathway and RAS/RAF/MEK/MAPK pathway, plays vital roles in several cellular processes, including inhibition of apoptosis, tumor growth, metastasis, and angiogenesis [69, 70]. It has been demonstrated that EGFR is overexpressed in CRC and it can be used as a prognostic biomarker in metastatic CRC [71, 72]. For this reason, mAbs have been designed to target EGFR for the treatment of CRC.

#### Cetuximab

Cetuximab, a chimeric mAb with approximately 152 kDa molecular weight, has a higher affinity to EGFR compared to its natural ligands, thereby inhibits the tumorigenesis effects of EGFR activation [73, 74]. Cetuximab, combined with chemotherapy, is the standard practice for first-line treatment of RAS wild-type metastatic CRC patients [75]. A phase II trial study revealed that once-weekly intravenous administration of cetuximab has modest anti-tumor activity and is well-tolerated in patients with chemotherapy-refractory CRC [76]. Although weekly administration of cetuximab is the standard protocol in patients with metastatic CRC, the results of a meta-analysis study indicated that biweekly administration of cetuximab instead of weekly administration shows equivalent efficacy and safety [77].

#### Panitumumab

Panitumumab, a fully human mAb against EGFR, was approved for the treatment of metastatic CRC in combination FOLFIRI and FOLFOX in the first- and second-line setting [78]. Binding of panitumumab to EGFR leads to internalization of the receptor, induction of apoptosis, inhibition of cell growth, and decrease in the production of VEGF and interleukin 8 [79, 80]. A randomized phase III study revealed that using panitumumab combined with FOLFOX4 significantly improved OS in patients with wild-type KRAS metastatic CRC [81]. It has been shown that testing KRAS mutation is critical in the administration of panitumumab to CRC patients, whereas KRAS mutations act as a predictor of resistance to therapy with panitumumab [82].

#### Necitumumab

High affinity of necitumumab, a fully humanized mAb, for EGFR inhibits phosphorylation of the EGFR and subsequent downstream signaling, leading to inhibition of tumor cell proliferation and tumor growth [83]. A phase II trial demonstrated that using 800 mg necitumumab every 2 weeks combined with chemotherapy (modified FOLFOX6) was active with manageable toxicity in metastatic CRC patients [84].

### Immune Checkpoint Inhibitors

Naïve T-cells require two signals to respond to tumor antigens and activation: The first one comes from the binding of T-cell receptors (TCRs) to complexes of an antigen presented on a major histocompatibility complex (MHC) class I molecules on the surface of a antigen presenting cell (APC)/tumor cell which is insufficient for activation of T-cell. The second signal that completes T-cell activation and proliferation occurs by binding of co-stimulatory receptor (CD28) on T-cell to B7 proteins on the APC/tumor cell [85]. Alongside the co-stimulatory receptors, T-cells also express co-inhibitory receptors, such as programmed cell death protein 1 (PD-1) and cytotoxic T-lymphocyte-associated antigen 4 (CTLA-4), which suppress T-cell function following binding to B7 and PD-1 ligand 1 or 2 (PD-L1 or PD-L2), respectively [86].

Immune checkpoint inhibitors (ICIs) which target co-inhibitory receptors or their ligands have revolutionized cancer therapy in several cancers. Anti-PD-1 and anti-PD-L1 antibodies directly block the PD-1/PD-L1 axis as well as the direct immune rejection of tumors through antibody-dependent cellular cytotoxicity (ADCC), whereas anti-CTLA-4 inhibitors allow free ligation of B7 to CD28 and enhance co-stimulatory signals of T-cells by directly blocking the receptor [87].

#### Pembrolizumab

Pembrolizumab, a humanized mAb against PD-1, was approved by the FDA in 2014 for preventing interaction between PD-1 and PD-L1/2 from restoring the immune responses [88]. A phase Ib study conducted by O’Neil et al*.* revealed that 24% of patients with CRC express PD-L1 in which treatment with pembrolizumab showed a manageable safety profile [89]. A phase 2 clinical trial demonstrated that 10 mg/kg intravenously administration of pembrolizumab is more efficient in mismatch repair–deficient (dMMR) CRC patients compared with mismatch repair–proficient CRC patients [90]. In another study, Dung et al*.* reported that once every 3 weeks administration of pembrolizumab to patients with dMMR/microsatellite instability-high (MSI-H) CRC provides durable anti-tumor activity and reasonable safety [91]. It has been shown that mismatch repair-deficient tumors strongly express immune checkpoint ligands, indicating that their microenvironment is resistant to tumor elimination [92].

#### Nivolumab

Nivolumab, a fully human mAb with a molecular weight of 146 kDa, targets PD-1 to block its interaction with PD-L1 and PD-L2 and subsequently avoiding PD-1 mediated inhibition of anti-tumor responses [93]. According to a phase I clinical trial by Yamamoto et al*.* in patients with solid tumors, nivolumab safety was reasonable at doses of up to 20 mg/kg [94]. Overman et al*.* conducted a multicentric phase II trial using nivolumab monotherapy and the combination of nivolumab with ipilimumab for the treatment of metastatic CRC patients with dMMR/MCI-H. They reported that the combination regimen improved outcomes, including disease control rate for 12 weeks or longer (80%), ORR (55%), and 12-month OS (85%). They also demonstrated that the treatment-related adverse effects in CRC patients were manageable [95].

#### Atezolizumab

Atezolizumab, a fully humanized mAb, targets PD-L1 and blocks its interactions with PD-1 and B7.1 (CD80) to restore the anti-tumor immune responses [96]. Antoniotti et al*.* designed a phase II clinical trial to assess the efficacy of FOLFOXIRI /bevacizumab regimen in combination with atezolizumab for the treatment of metastatic CRC. They indicated that this combination regimen is safe for patients without grade 4 adverse events [97]. A phase III randomized clinical trial, IMblaze 370, was conducted on metastatic CRC patients using atezolizumab (1200 mg), regorafenib (160 mg), and atezolizumab (840 mg) plus cobimetinib (60 mg). The results showed that the median OS in the atezolizumab, regorafenib, and atezolizumab plus cobimetinib groups was 7.10, 8.5, and 8.87 months, respectively. Moreover, the combination therapy showed more adverse events than monotherapy with atezolizumab. Overall, this trial did not demonstrate significant improvements and safety in patients with microsatellite-stable metastatic CRC receiving the combination therapy [98].

#### Ipilimumab

Ipilimumab, a fully humanized mAb against CTLA-4, was approved by FDA for the treatment of melanoma in 2011 [99]. The positive results of ipilimumab plus nivolumab was demonstrated in the treatment of renal cell carcinoma, melanoma, and metastatic CRC [100]. A multicentric phase II study, CheckMate 142, is designed to assess the safety and efficacy of nivolumab in combination with low-dose ipilimumab in MSI-H/dMMR CRC patients. The results of the study revealed that the combinational regimen is safe and the adverse events were generally low grade. However, the occurrence of adverse events did not affect the efficacy of the treatment with nivolumab in combination with low-dose ipilimumab. Thus, combinational therapy targeting both immune checkpoints (PD-1 and CTLA-4) provides promising [101].

#### Tremelimumab

Tremelimumab is a fully human mAb against CTLA-4, which blocks inhibitory signals of CTLA-4, leading to immune activation [102]. Chung et al*.* evaluated the efficacy of tremelimumab in patients with refractory CRC. They reported that intravenously administration of tremelimumab (15 mg/kg) every 90 days, as a monotherapy, had not clinically benefit. However, the manageable toxicity and mechanism of action suggest that a combination of tremelimumab with other therapeutic agents may be effective [103].

### Other targets

In addition to the mentioned targets, other mAbs also have been designed to target different pathways in CRC. For example, tigatuzumab, a fully human mAb targeting Apo2L/TRAIL death receptor DR5, was applied in combination with mFOLFOX6 and bevacizumab in patients with metastatic CRC. This combinational regimen showed evidence anti-tumor activities with no dose-limiting toxicities [104]. A phase II study using robatumumab, a fully human anti- insulin-like growth factor 1 receptor (IGF-1R), revealed that patients with refractory CRC might show transient response to robatumumab [105]. Other mAbs also were tested for the treatment of CRC patients, including nimotuzumab, drozitumab, i-huA33, MNRP1685A, KRN330, tigatuzumab, and RG7212 [106].

## Challenges of mAbs

Some factors are determinants in comparing different clinical trial results and clinical outcomes of mAbs, including sample size, genetic differences between the study populations, and drug dosage. For instance, it was mentioned that KRAS mutations act as a predictor of resistance to therapy with panitumumab [82] or microsatellite instability acts as a predictive marker in treatment with immune checkpoint inhibitors [90, 107]. Furthermore, determining fixed dosing of anticancer therapies offers considerable advantages, including lower costs, reduced dose preparation errors, and ease of dose preparation [108]. Considering the pharmacokinetic data, such as half-life, allows administration scheduling of each mAb and optimal anti-tumor effects [77]. The toxicity of mAbs and their combination with toxicity agents is another factor determining the success of mAb. Although the toxicity in using anti-angiogenesis agents in combination with chemotherapy is manageable, an increase in toxicity profile is associated with the usage of chemotherapy agents. However, there are some toxicities related to anti-angiogenesis agents. For example, hypertension and proteinuria are the most common side effects of bevacizumab [109, 110].

There are some challenges in the designing, manufacturing, formulation, and stabilization of mAbs. For example, posttranslational modifications of amino acids such as methionine oxidation, aspartic acid isomerization, and asparagine deamidation can lead to a lack of potency and/or heterogeneity if they are involved in the recognition and interaction with the target. Moreover, tryptophan can induce molecular aggregation, resulting in a lack of solubility and immunogenicity [111]. Thus, considering these biophysical properties will help to produce favorable therapeutic mAbs.

Protein A chromatography is known as the golden standard for mAb purification. The main challenge in using this technology is related to resins high costs and their low lifetime compared to others. Furthermore, protein A ligands cannot bind all types of IgG and other therapies such as antibody fragments without the Fc region. Different ligands such as a combination of protein A and new affinity ligands and protein L could develop at the commercial level in mAb purification to address this challenge [112]. Also, applying harsh conditions such as pH and salt to eliminate viral contaminations can lead to antibody instability and loss [113, 114].

Another consideration in mAbs is their formulation and clinical use. Non-invasive administration, including nasal, pulmonary or oral, exposes therapeutic mAbs to enzymatic and chemical degradation in the gastrointestinal (GI) tract. Due to relatively large molecular weight and polar surface charge, the bioavailability of therapeutic mAbs through non-invasive routes is poor. Several strategies, including nanoparticles, liposomes, and microencapsulation, have been developed to control mAb release and stability, prolonging their half-life. Moreover, these nanocarriers can increase tumor penetration of mAbs [115].

## CAR-T cell therapy

### Overview of CAR-T cell

**Fig. 2:**
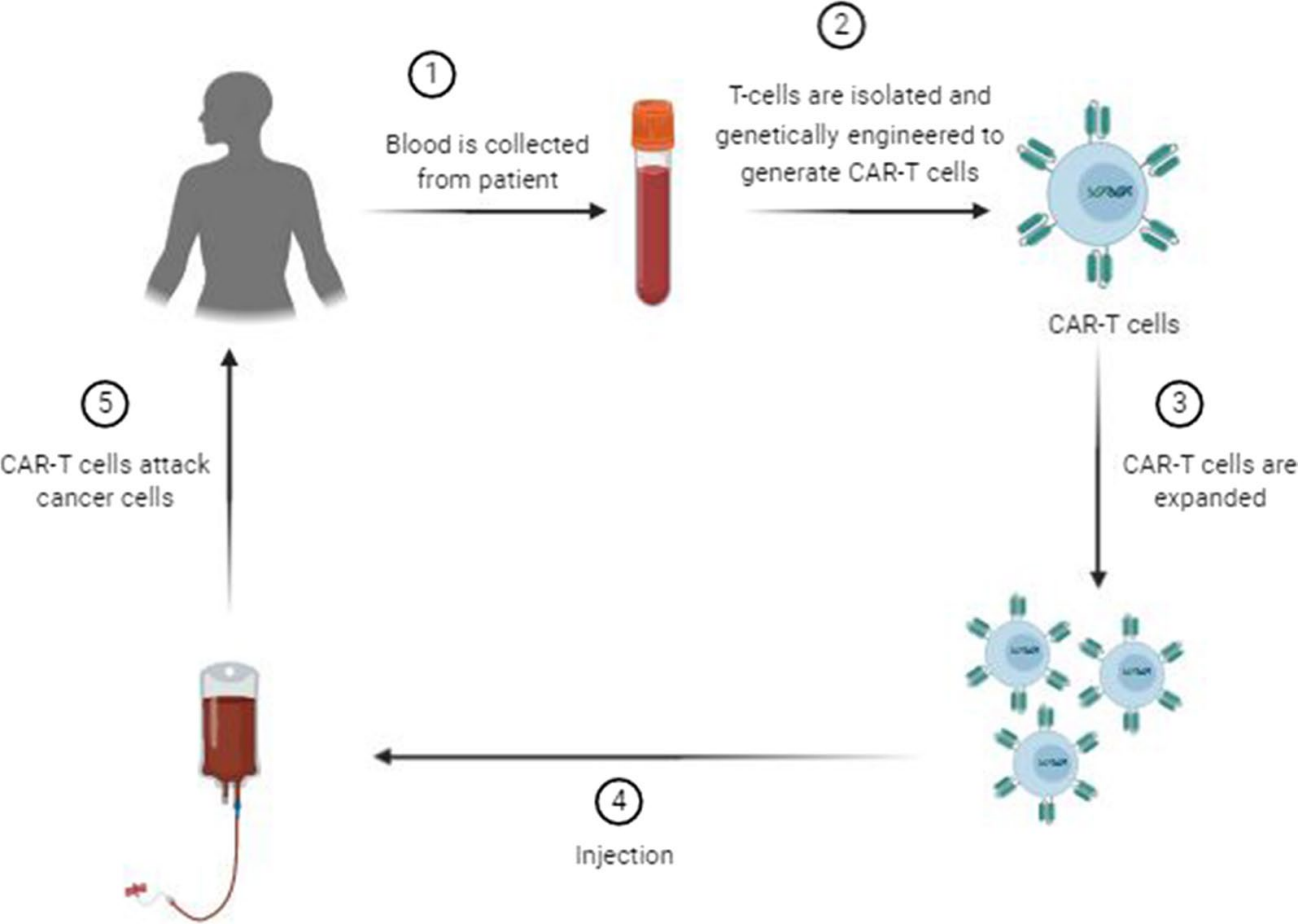
Overview of CAR-T cell therapy. The blood of the patient is collected and T cells are isolated. T cells are then genetically altered to express specific receptors for binding to certain targets on the cancer cells. The generated CAR-T cells are cultivated and expanded in vitro. Finally, CAR-T cells are infused into the patient’s bloodstream to kill the tumor cells

Adoptive T-cell therapy (ACT) has long been used for cancer therapy in which adaptive T-cells are transferred into the patients. The first ACT was developed for melanoma by isolation and expansion of tumor-infiltrating lymphocytes (TILs) from patients in the 1990s [116]. In TIL therapy, tumor biopsy is fragmented and plated in the presence of interleukine-2 (IL-2) for 2–4 weeks. The cultured TILs are selected and obtained according to the T-cell phenotype and reactivity to tumor cells for infusion into the patient [117]. Despite promising beneficial effects, many hurdles limit immunotherapy based on TILs. Due to TILs limited numbers in many tumors, their isolation is a time-consuming procedure [118]. Although the extracted TILs are tumor-specific, a remarkable portion of extracted cells has suppressive functions rather than anti-tumor activity [119]. Furthermore, immunotherapy with TILs is MHC-restricted and based on the recognition of TAAs by MHC, while the majority of tumor cells, mostly solid tumors, downregulate the expression of MHC [120]. Thus, genetically engineered T-cells have been emerged to overcome these hurdles. Figure 2 shows the generation process of CAR-T cells.

**Fig. 3:**
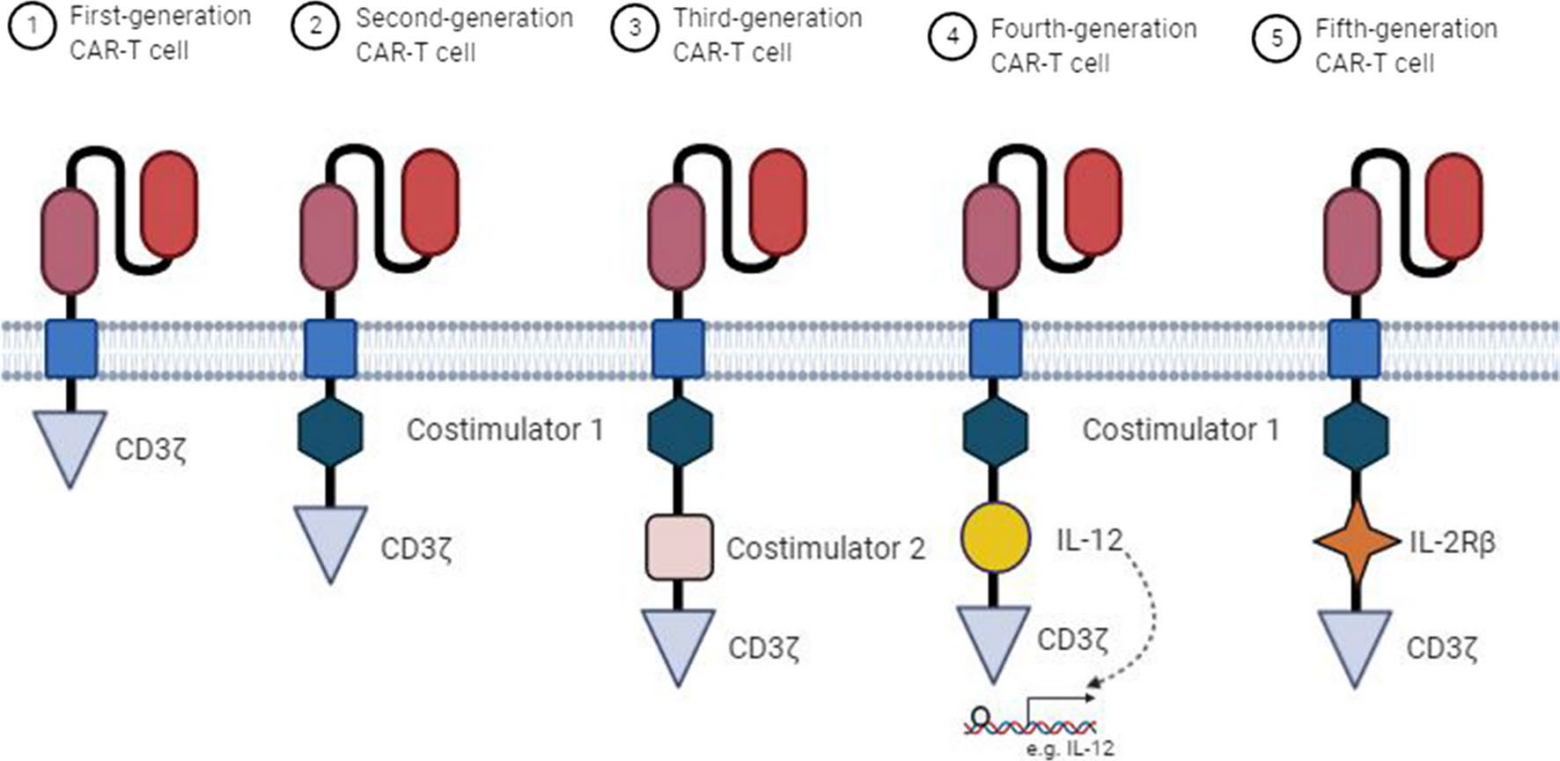
Five generations of CAR-T cells. The first-generation CAR-T cells only contain one intracellular signal domain CD3ζ. The second-generation CARs consist of a co-stimulatory domain, including 4-1BB (CD137) or CD28, whereas the third-generation ones have two co-stimulatory domains. The fourth-generation CAR-T cells, based on the second-generation CARs, can induce cytokine production. The fifth-generation CAR-T cells are also based on the second-generation CARs, containing intracellular domains of cytokine receptors, such as IL-2Rβ chain fragment

Chimeric antigen receptors (CARs) are bioengineered and hybrid of T-cell and antibody receptors composed of 4 distinct regions, including an extracellular domain, a hinge, a transmembrane (TM) domain, and an intracellular signaling domain (endodomain). The extracellular domain which recognizes antigen is a single-chain fragment variant (scFv) contains the variable light and heavy chain regions of an antibody separated via a flexible linker and is connected to the transmembrane domain by a hinge (spacer) [121]. The TM domain, a hydrophobic alpha-helix structure, guarantees the stability and expression of the receptor [121, 122]. The endodomain undergoes conformational changes following antigen recognition, triggering downstream signaling pathways to induce immune responses [123]. According to the composition and structure of the endodomain, five generations of CAR-T cells have been developed (Fig. 3). The first-generation CARs contain only CD3ζ intracellular domain. Owing to a lack of co-stimulatory and interleukin signals, the anti-tumor activity of them is limited [124]. To enhance their functions, the scientists added a co-stimulatory domain, including 4-1BB (CD137) or CD28, and developed the second-generation CAR-T cells [125]. A third-generation of CARs contains two co-stimulatory domains, such as both 4-1BB and CD28 [126]. The fourth-generation CAR-T cells, T-cells redirected for universal cytokine-mediated killing (TRUCKs), are designed based on the second-generation CARs which contain additional domains for cytokine secretion, such as IL-2. The fifth-generation or next-generation CARs are also based on second-generation CARs which include intracellular domains of cytokine receptors, such as IL-2Rβ [127].

### CAR-T cell therapy for CRC

CAR-T cell therapy is becoming an interesting candidate in the treatment of cancer. Although most CAR-T cell therapy studies focus on the treatment of hematologic malignancies, such as lymphoma, with targeting CD19 antigen, demand for their application in the treatment of solid tumors in clinical trials is high. Different TAAs were translated into clinical trials for the treatment of CRC, including carcinoembryonic antigen (CEA), EGFR, mesothelin (MSLN), mucin 1 (MUC1), Natural killer group 2 member D (NKG2D) and its ligand, human epidermal growth factor receptor 2 (HER2), c-met, and CD133 (Table 2).

**Table 2:** CAR-T cell therapy under clinical trials for CRC

Target	Phase	Sponsor	NCT number
CEA	eI^*^	Ruijin Hospital	NCT04513431
EGFR	I/II	Shenzhen Second People's Hospital	NCT03152435
EGFR/IL12	I	Shenzhen Second People's Hospital	NCT03542799
αPD1/MSLN	eI	Shanghai Cell Therapy Group Co.,Ltd	NCT04503980
NKG2D	I	Celyad Oncology SA	NCT03692429
MUC1	I/II	PersonGen BioTherapeutics (Suzhou) Co., Ltd	NCT02617134
CEA	I/II	Shanghai GeneChem Co., Ltd	NCT02959151
NKG2D	I	Celyad Oncology SA	NCT03370198
NKG2D	I	Celyad Oncology SA	NCT03310008
NKG2DL	I	The Affiliated Nanjing Drum Tower Hospital of Nanjing University Medical School	NCT04550663
CEA	I/II	Chongqing Precision Biotech Co., Ltd	NCT04348643
CEA	I	Southwest Hospital, China	NCT02349724
HER-2	I/II	Zhi Yang	NCT02713984
CEA	I	Sorrento Therapeutics, Inc	NCT03682744
C-met	I/II	Shenzhen BinDeBio Ltd	NCT03638206
HER-2	I	Baylor College of Medicine	NCT03740256
NKG2DL	I	CytoMed Therapeutics Pte Ltd	NCT04107142
CD133	I/II	Chinese PLA General Hospital	NCT02541370

* Early phase I

### CEA

CEA, a glycoprotein belonging to the carcinoembryonic antigen cell adhesion molecules (CEACAM) family, is produced in the GI tract of humans in the early stages of embryonic development, whereas its expression decreases before birth and remains at a low level in the adult [128]. However, CEA expression is elevated in CRC and tissue CEA overexpression is associated with poorer prognosis and can act as a tumor marker in CRC [129]. Thus, CEA is an attractive target of CRC immunotherapy.

Chi et al*.* investigated the anti-tumor efficacy of CEA-specific CAR-T cell, a second-generation CAR in which 4‐1BB acted as a costimulatory domain, in combination with recombinant human IL‐12. They indicated that IL-12 markedly increases proliferation, activation, and cytotoxicity of CEA‐CAR‐T cells following treating CEA-positive HT‐29 cells. Combination with IL-12 enhances the expression levels of IFN‐γ, IL-2, CD25, and CD69, as markers for activation of CAR-T cells. Intravenous administration of 1 × 10^7^ CEA‐CAR‐T cells in combination with 1500 U/mouse IL-12 showed stronger anti-tumor function compared with CEA‐CAR‐T cell administration in mice model. This combination regimen increased the persistence of CEA‐CAR‐T cells and enhanced secretion of IFN‐γ, TNF‐α, and IL-2 [130]. Blat et al*.* designed a CEA-targeting CAR regulatory T cells (Tregs) to suppress colitis-associated CRC. They showed that CEA-targeting CAR Tregs could reduce the progress of induced colitis toward CRC in a mouse model and decrease tumor burden [131]. In a phase I clinical trial, Zhang et al*.* investigated the safety and efficacy of CAR-T cell therapy in the treatment of CEA-positive CRC patients. They designed two CARs: a second-generation CAR with CD28 signaling domain and a third-generation CAR with CD28 and CD137 domains. Of the ten patients, two patients indicated tumor shrinkage and seven patients had stable disease in which two patients showed stable condition for more than 30 weeks after CAR-T cell therapy. The serum analysis revealed that CEA levels declined in most of the patients. In high-dose receiving, the persistence of CARs was observed. Furthermore, the cytotoxicity and cytokine secretion of the third-generation CARs was not better than the second-generation ones [132].

### GUCY2C

Guanylyl Cyclase C (GUCY2C), a membrane-bound receptor, is regularly expressed by apical surfaces of the intestinal epithelium and in the brain [133]. Binding of cognate ligands to the GUCY2C produces cGMP as a second messenger, leading to regulation of colon homeostasis, obesity, and tumorigenesis [134]. It has been shown that GUCY2C is universally overexpressed in the primary and metastatic CRC [135, 136], making it a tempting immunotherapy target.

Magee et al*.* designed a third-generation GUCY2C-specific CAR-T cell composed of scFv, the CD8α hinge region, the TM and endodomain of CD28, and the endodomain of CD137 and CD3ζ and tested its efficacy in a metastatic CRC mice model. They found that GUCY2C CAR-T cells reduced the number of metastatic tumors in mice's lungs and the treated-mice showed reduced morbidity and improved survival without inducing autoimmunity. Moreover, treatment with GUCY2C CAR-T cells did not show toxicity and accumulation in the mice intestine [137]. This group also demonstrated that the protection of GUCY2C CAR-T cells against lung metastasis is long-term [138].

### NKG2D

NKG2D is regularly expressed on the natural killer (NK) cells, CD8 + T-cells, some CD4 + T-cells, and γẟ T-cells which provides activating and costimulatory signals [139]. In humans, NKG2D interacts with 8 NKG2D ligands (NKG2DLs), including MHC class I-related chain A and B (MICA and MICB) and six unique long 16 (UL16)-binding proteins (ULBP1–6). Although NKG2DLs are undetectable or at low levels on healthy tissues, it is upregulated upon infection, DNA damage, and cell transformation [140, 141]. Binding of NKG2DLs to NKG2D activates immune cells, triggering cells' proliferation, producing proinflammatory cytokines, and eliminating target cells.

Deng et al*.* constructed a third-generation CAR composed of the CD8α signal sequence, the extracellular region of human NKG2D, the CD8α hinge, the TM and endodomain of CD 28, and the endodomains of CD3ζ and CD137. NKG2D CAR-T cells indicated cytotoxicity against human CRC in vitro in a dose-dependent manner and secreted high levels of IFN-γ and IL-2. Administration of 1 × 10^7^ NKG2D CAR-T cells markedly inhibited tumor growth, reduced tumor size, and prolonged survival in a xenograft CRC mice model without any severe side effects in vital organs [142]. In another study, Xiao et al. used an RNA electroporation approach to provide NKG2D RNA for NK cells and assessed its anti-tumor functions. NKG2D RNA CAR therapy remarkably enhanced the cytolytic function of NK cells against tumor cells in vitro and significantly reduced tumor progression and prolonged survival time in a CRC mice model. Treatment of patients with metastatic CRC using NKG2D RNA CAR reduced ascites generation and tumor cell number in ascites samples [143]. Zhang et al*.* constructed NKG2D CAR T cells to target Rae1, an NKG2DL, for inhibiting tumor vasculature. They showed that NKG2D CAR T cells reduced tumor angiogenesis in a colon cancer mice model [144].

### Other targets

Wei et al. a third-generation CAR-T cell for targeting epithelial cell adhesion molecule (EpCAM) which is overexpressed in different cancers. EpCAM CAR-T cells were able to show cytotoxicity against EpCAM positive CRC cells and secrete cytokines, such as IFN‐γ and TNF‐α. This adaptive therapy also significantly suppressed tumor growth and formation in a xenograft model without systemic toxicity [145].

Tumor-associated glycoprotein (TAG)-72 is another TAA that has been targeted for the treatment of CRC using CAR-T cells. TAG-72 is oncofetal mucin highly expressed in most of the human epithelial adenocarcinomas and its expression is mainly restricted to tumor cells [146]. Hege et al*.* constructed a first-generation TAG-72 CAR-T cell and tested its safety and efficacy in patients with metastatic CRC. They demonstrated that TAG-72 CAR-T cell therapy is relatively safe, whereas their persistence is limited owing to the lack of costimulatory domains [147].

### Challenges of CAR-T cells

CAR-T cell therapy is faced with various challenges in the treatment of solid tumors. For example, physical barriers, including the surrounding stroma and cells, hinder the sufficient infiltration of CARs to the tumors. To overcome these barriers, Wang et al*.* designed FAP-CAR T cells to target fibroblasts in the tumor microenvironment (TME), leading to reduce the number of tumor fibroblasts and inhibition of tumor growth [148]. Targeting heparan sulfate proteoglycans, as a member of extracellular matrix (ECM) which limits cells homing to the tumors, with heparanase (HPSE)-expressing CARs also overcomes the physical barrier and improves CARs infiltration to the tumor [149] (Fig. 1).

The other concern in using CAR-T cells is their on-target/off-tumor toxicity. Since most tumor antigens are found both on cancerous cells and normal tissues, constructed CARs cannot distinguish the normal ones. Thus, identifying specific tumor antigens always is the CAR-T cell therapy challenge. For instance, a case-study of a patient with metastatic cancer expressing HER-2 revealed that antigen-specific CARs could result in organ dysfunctions, rapid respiratory distress, and death. Accumulation of anti-ERBB2 CARs in the lung and recognizing ERBB2-expressing normal cells and subsequent release of IFN-γ and TNF-α led to pulmonary toxicity and edema [150]. This toxicity is associated with the release of cytokines, also known as cytokine release syndrome. In another study, it was reported that targeting CEA in patients with colon cancer by CAR-T cells led to severe transient colitis because of CEA recognition in normal intestinal tissue [151] (Fig. 2).

Immunosuppressive TME also reduces CARs efficacy. TME consists of immune cells, including myeloid-derived suppressor cells (MDSCs), Tregs, and tumor-associated macrophages (TAMs), and molecular factors, including checkpoint inhibitory proteins, IL-10, and TGF-β, that inhibit anti-tumor functions of CAR-T cells [152]. There is evidence that blockade of the inhibitory pathways can augment CAR-T cell therapy. For example, the combination of an anti-PD-1 antibody with HER2 CAR T cells significantly enhanced tumor eradication in a mouse model [153] (Fig. 3).

## Conclusions

Standard treatments for CRC, including surgery, radiotherapy, and chemotherapy, have many side effects owing to their non-specificity toward normal cell, leading to toxicity. Thus, alternative and safety treatments are crucial for CRC patients. In this regard, vaccines, cytokines, adjuvant, and neoadjuvant therapy to stimulate immune responses against tumor antigens have been developed as immunotherapy agents against CRC. Taken together, immunotherapy using both mAbs and CAR-T cells are effective with minimal side effects and toxicity. Preclinical and clinical studies demonstrated that CRC cells are vulnerable to specific mAbs. Furthermore, clinical trials using CARs could have promising therapeutic outcomes in patients with CRC. Although some challenges and concerns are using both mAbs and CAR-T cells, combinational therapies will improve the anti-tumor effects and optimize their functions.

## Data Availability

Not applicable.

## Abbreviations

CRC: Colorectal cancer; mAbs: Monoclonal antibodies
CAR-T cells: Chimeric antigen receptor T cells
TAAs: Tumor-associated antigens
TME: Tumor microenvironment
VEGF: Vascular endothelial growth factor
EGFR: Epidermal growth factor receptor
IGF-1R: Insulin-like growth factor 1 receptor
HER2/neu: Human epidermal growth factor receptor-2
MUC5AC: Mucin 5AC
DR5: Death receptor
CTLA-4: Cytotoxic T-lymphocyte associated protein 4
PD-L1: Programmed death-ligand 1
PlGF: Placental growth factors
TCR: T cell receptors
MHC: Major histocompatibility complex
APC: Antigen-presenting cell
ADCC: Antibody-dependent cellular cytotoxicity
GI: Gastrointestinal
ACT: Adoptive T-cell therapy
TILs: Tumor-infiltrating cells
TM: Transmembrane
scFv: Single-chain fragment variant
CEA: Carcinoembryonic antigen
MSLN: Mesothelin
MUC1: Mucin 1
NKG2D: Natural killer group 2 member D
Tregs: Regulatory T cells
GUCY2C: Guanylyl cyclase C
TAG-72: Tumor-associated glycoprotein-72
